# Priorities and Recommendations for Using Artificial Intelligence (AI) to Improve Equid Health and Welfare

**DOI:** 10.3390/ani16071082

**Published:** 2026-04-01

**Authors:** Philippa L. Young, Robert Hyde, Janet Douglas, Sarah L. Freeman

**Affiliations:** 1 School of Veterinary Medicine and Science, University of Nottingham, Loughborough LE12 5RD, UK; robert.hyde4@nottingham.ac.uk (R.H.); sarah.freeman@nottingham.ac.uk (S.L.F.); 2 World Horse Welfare, Anne Colvin House, Norwich NR16 2LR, UK; janetdouglas@worldhorsewelfare.org

**Keywords:** artificial intelligence, barriers, concerns, consensus, Delphi, equid, horse, priorities, solutions, welfare

## Abstract

Artificial Intelligence (AI) is being increasingly used for equid health and welfare. This study aimed to establish consensus on where and how AI should be developed to achieve maximum benefit in this field. A workshop involving 41 stakeholders generated statements about current welfare concerns, areas for AI development, and barriers and solutions to AI use. Statements were circulated through Delphi surveys (acceptance set at 75% agreement). One-hundred-and-six statements reached agreement. Ethological needs not being met and poor equid management practices were key welfare concerns. Participants identified that insufficient owner/carer knowledge and understanding were important factors contributing to welfare concerns. Priority areas for AI development included assessment of equid wellbeing, as well as individual and population-level monitoring. Barriers included limited understanding of both equine behaviour and AI, biased, unethical, or insufficient data collection, difficulties developing accurate models, challenges to validation, and uncertainty around interpretation. Proposed solutions included development of evidence-based, unbiased AI systems, following best practice guidelines, requiring approval/regulation of AI tools, collaboration, and education of AI users. This is the first study to identify stakeholders’ opinions about where AI is likely to have the greatest benefit for equids, potential barriers, and solutions. The findings should be used to prioritise funding and development.

## Simple Summary

Artificial intelligence (AI) tools are increasingly being used to understand and improve the health and wellbeing (welfare) of horses, ponies, donkeys, and their hybrids (‘equids’). However, it is important that these tools support the right areas and avoid potential pitfalls. This study gathered experts’ opinions on where and how AI tools should be developed and used to improve equid welfare. Forty-one people from across the equid industry attended a workshop where they identified current welfare problems for UK equids, areas where AI tools should be developed, and problems with/solutions to the use of AI. Participants then rated the ideas in online surveys, agreeing on 106 of them. Key welfare problems involved equid management and failure to meet their needs. Areas where AI tools should be developed included measuring equid wellbeing, monitoring individual equids, and monitoring equid populations. Data collection, AI model development, and model validation were among the problems identified. Suggested solutions included developing evidence-based, unbiased AI systems, education, regulation, and working together. This is the first study to identify stakeholders’ opinions about which AI tools would be most beneficial for equid welfare, potential problems, and solutions. Future funding and development of AI tools should be guided by these results.

## 1. Introduction

Artificial intelligence (AI) offers innovative strategies to improve equid welfare—which includes health [1]. AI can be defined as computer systems capable of human-like activities, such as the ability to perceive, solve problems, make decisions, and learn [2]. Common subsets of AI include machine learning (computer systems that recognise patterns in existing data, allowing them to predict the outcome from new data), computer vision (computer systems that take in image data and provide interpretations such as object recognition), and generative AI (computer systems that create novel content such as text or images).

Recent reviews demonstrate the breadth of AI’s applications in the animal welfare field. These include identifying animal behaviour and emotion [3], improving animal management [3], and detecting disease [4]. The work here focused on the welfare of horses, ponies, donkeys, and their hybrids (hereafter referred to as “equids”) in the United Kingdom (UK). In the equid industry, AI has already been used to monitor behaviour, detect lameness, assist in veterinary examinations, and predict prognosis for colic cases [5]. The promise of AI technologies is reflected in their rapid growth: interest in AI has been increasing rapidly since 2014 [6], and the UK has seen an 85% increase in the number of AI companies between 2022 and 2024 [7].

In the current study, our interest is in the ability of AI technologies to positively influence equid health and welfare—and here it faces limitations. First of all, there is currently no general regulation of AI in the UK [8] and consideration of the risks and benefits of AI within the veterinary sector is at an early stage, with no regulation in place [9]. The industry must also overcome the ethical and physical challenges [10] and risks [11] associated with the use of AI.

It is likely that the AI field would deliver maximal benefit for equid health and welfare if its development were targeted towards the areas of greatest need. The work reported here used a Delphi process to establish stakeholder opinion on where and how AI tools could be developed and used to deliver the greatest benefits for equid health and welfare. The study’s aims were to (1) identify current welfare priorities for UK equids, (2) identify priority areas for AI tools to be developed, (3) identify barriers associated with the development and use of AI tools, and (4) identify possible solutions to overcome these barriers. This work is particularly important at this time, when both the AI industry and concern for the welfare of UK equids are growing. Ultimately, the work will inform the development, use, and uptake of AI tools, thereby potentially improving the welfare of equids in the UK.

## 2. Materials and Methods

The study was approved by the University of Nottingham’s Research Ethics Committee (reference number: 4239 181024) and all participants completed a consent form before being involved in the research.

### 2.1. Stakeholder Recruitment

Key stakeholder groups were identified, and each group was assigned a target number of participants. Stakeholder groups comprised veterinarians, equid owners/carers/coaches, welfare researchers, welfare advisers, regulatory bodies, AI companies, and Animal Welfare Research Network (AWRN) members. Prospective participants were identified and invited to participate by convenience sampling. This focused on individuals within/in close proximity to the University of Nottingham, those who had contributed to or participated in previous welfare or stakeholder studies, and those who were considered to be influential or representative within their stakeholder group. For larger research groups, charities, or regulatory bodies, invitations were sent to key contacts within each group. These contacts then selected the most relevant and available person within that team. Participants were also invited via notifications distributed through the AWRN newsletter. The maximum number invited for each stakeholder group was 10, except for regulatory bodies and AI companies, where the maximum number invited was 5 for each group. Inviting AWRN members was a requirement of the funding for this study, and these individuals were included in the welfare research group (combined maximum of 10). If invitees declined or withdrew, another stakeholder was invited where possible. Some attendees fitted into more than one stakeholder group (e.g., veterinarian and welfare researcher). Participants were allocated to ensure representation from each stakeholder perspective at each table (e.g., a participant could represent more than one stakeholder view).

### 2.2. Workshop

An in-person workshop was held at The School of Veterinary Medicine and Science, University of Nottingham, England on 29 January 2025. Stakeholders were placed into mixed groups with a facilitator and a notetaker. The workshop was structured into three sessions. Each session involved an introductory talk, with or without associated presentations, followed by facilitated discussion. Each facilitator was provided with a written guide to help structure the discussions and keep the conversations on topic. Perspectives from all stakeholders were sought and recorded. Different or conflicting opinions were considered, and if the group did not agree, then both points were recorded and taken forward to the online consensus survey. The aims of each session, details of the introductory talks and presentations, and discussion points are summarised in Table 1.

### 2.3. Delphi Surveys

The notes from all workshop groups were collated and participants’ ideas were extracted. Duplicate ideas and those which were too broad or unclear to allow generation of a statement were excluded. Ideas not relevant to equid welfare or AI within the context of the study (e.g., predictions about the future of the AI industry, or discussions about possible financial models of AI tools) were excluded. The remainder (including any competing or contradictory ideas) were grouped into themes and sub-themes.

The ideas were transformed into statements (including being reworded to add clarification, definition, or explanation, as needed; PY), checked and amended by another researcher (SF), and compiled into a traditional Delphi survey [13] (PY and SF). The survey was built in Microsoft Forms and used 4- and 5-point Likert scales (plus an additional option: “Unsure/I don’t know”) to assess participants’ opinions on the statements. The Likert scales used were as follows:

- High priority; Medium priority; Low priority; Not a priority; Unsure/I don’t know.
- Very effective; Somewhat effective; Neither effective nor ineffective; Somewhat ineffective; Very ineffective; Unsure/I don’t know.
- Very concerned; Somewhat concerned; Neither concerned nor unconcerned; Somewhat unconcerned; Very unconcerned; Unsure/I don’t know.
- Strongly agree; Agree; Neutral; Disagree; Strongly disagree; Unsure/I don’t know.

In total, three rounds of the survey were generated. Each survey was sent to participants via e-mail, and they were given a minimum of ten days to complete it. Reminders were sent before the closing date for each round and an extension was offered. Results were downloaded into Excel (v16.100.1) for data cleaning and analysis.

The first round survey collected demographic data from participants (age, gender, level of education, role(s) in the equid industry, and familiarity with AI) and included a free-text box for comments at the end of each theme (‘section’). The second- and third-round surveys included a free-text box for comments at the end. These surveys were sent only to participants who had completed the previous round.

### 2.4. Analysis and Delphi Process

After each round had closed, duplicate responses from the same individual were removed and the ‘agreement’ with each statement was calculated. Agreement was defined as the proportion of respondents who selected one of the first two categories (as defined above) on the Likert scale for that statement (e.g., for the priority Likert scale, this was the proportion of respondents who chose ‘High priority’ or ‘Medium priority’).

Statements with at least 75% agreement were ‘accepted’ and removed from further rounds. Where possible, statements with between 25 and 75% agreement were amended based on participant feedback, reviewed by two researchers (PY and SF), and included in the next round of the survey. After round 1 only, statements with between 25 and 75% agreement for which the research team felt that participant feedback might be helpful for clarification/improvement of the wording were included, verbatim, in the second round, accompanied by a request for participant input. For all rounds, statements with between 25 and 75% agreement, which were considered to already be as clear as possible, were rejected and removed from any further rounds, as were all statements that received ≤ 25% agreement. In round 3, any statements which did not reach 75% agreement were rejected. In rounds 1 and 2, free-text comments were reviewed and new statements that arose from them were included in the next round.

Figures were generated in R (version 4.5.2) [14] using the RStudio interface (version 2025.9.2.418) [15] and the ‘plotly’ package (version 4.11.0) [16]. Statements with the same level of agreement were ranked based on the proportion of respondents who selected the higher of the two ‘agreement’ options on the Likert scale. Any remaining ties were ordered alphabetically.

## 3. Results

Of the 70 stakeholders who were invited to participate in the workshop, 41 attended. The most common stakeholder group represented by the workshop participants was veterinarians (Table 2). The other stakeholder groups represented were equid owners/carers/coaches, welfare researchers, welfare advisers, regulatory bodies, AI companies, and AWRN members.

The themes and sub-themes identified during the workshop are shown in Table 3.

### 3.1. Survey Participants

**Figure 1:**
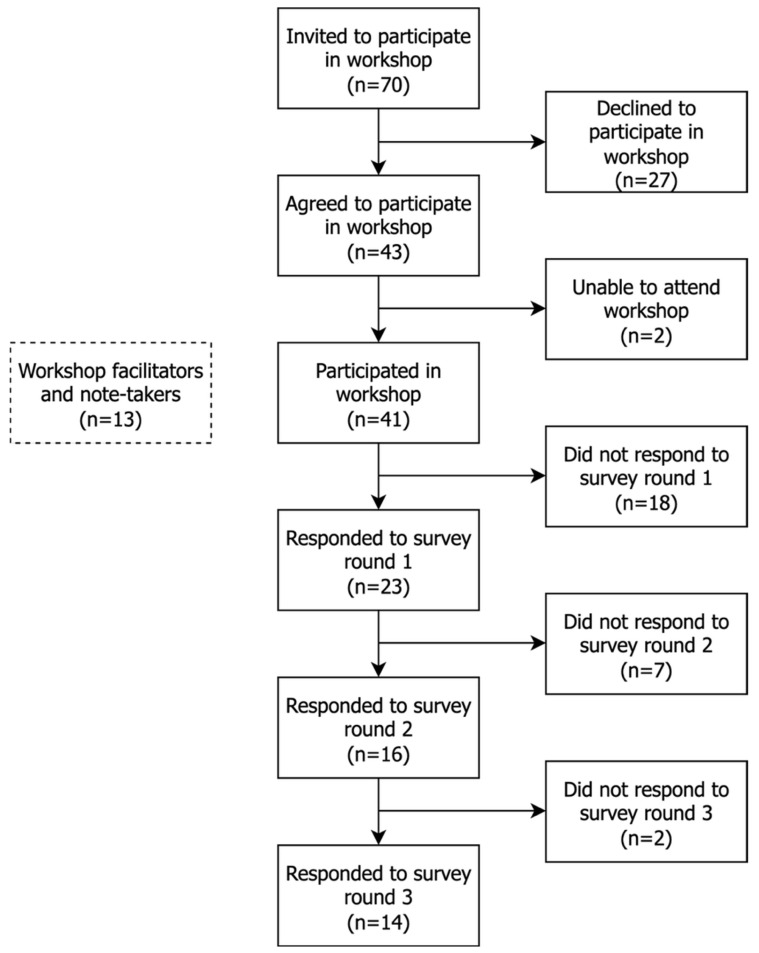
Flow chart representing the number of participants attending the in-person workshop and completing each round of the survey from a Delphi study exploring stakeholders’ opinions about equid health and welfare and the related use of AI.

Of the 41 stakeholders who attended the workshop, 23, 16, and 14 completed the first, second, and third surveys, respectively (Figure 1). One duplicate response was removed from the results of the first survey. As the survey was anonymised, data relating to which participants/stakeholder groups completed each round are not available.

The survey participants were predominantly female (78%, 18/23), with a modal age of 40–49 years (Table 4). They had a range of highest educational levels, with the mode being a PhD (52%, 12/23). Their involvement in equid health and welfare spanned all 11 roles listed in the survey (see Supplementary Materials). Participants could select multiple roles. The most commonly selected were as an owner/carer (78%, 18/23) followed by delivering public education (61%, 14/23), delivering higher education (57%, 13/23), and involvement in research (52%, 12/23) on equid health and welfare. The majority felt that they could either understand (44%, 10/23) or take part in (39%, 9/23) a conversation on AI.

### 3.2. Statements Generated from the Workshop and Delphi Process

**Figure 2:**
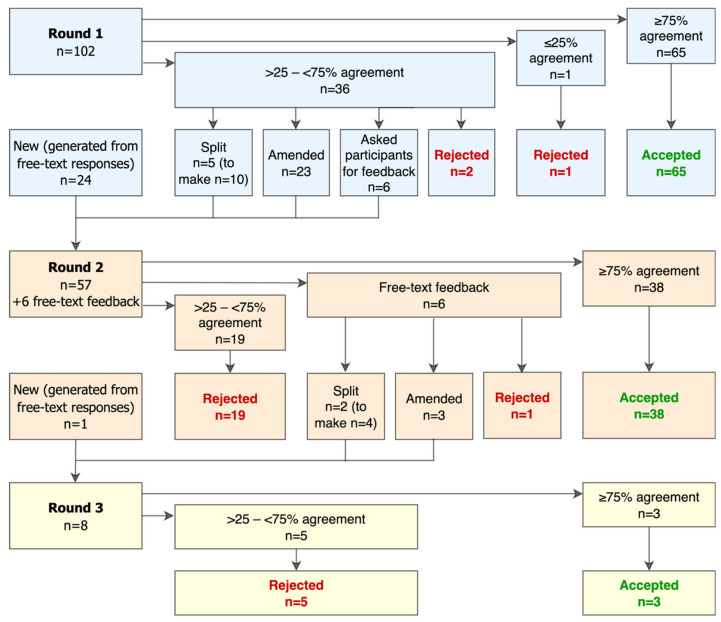
A flow chart showing the origin and outcome of the 134 statements generated throughout the Delphi process exploring stakeholders’ opinions about equid health and welfare and the related use of artificial intelligence.

A total of 134 statements were generated throughout the Delphi process (Figure 2). Of these, 106 were accepted and 28 were rejected.

### 3.3. Agreed Statements

#### 3.3.1. Welfare Priorities

**Figure 3:**
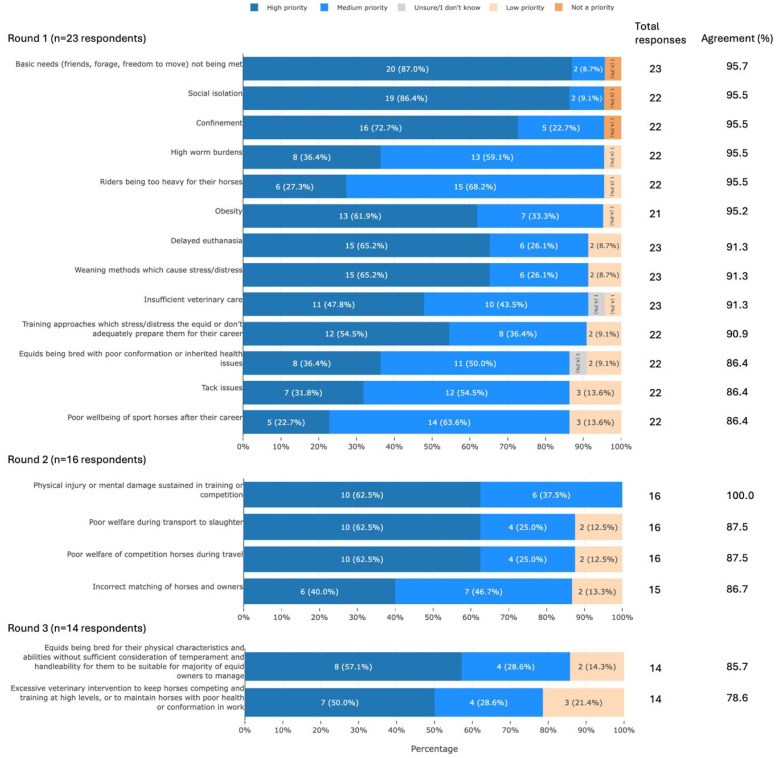
The 19 welfare priorities that met agreement, split by survey round and ranked by percentage agreement, from a Delphi study exploring stakeholders’ opinions about equid health and welfare and the related use of artificial intelligence. Within each statement, values may not sum to 100.0% due to rounding. Statements have been shortened for brevity. The full statements are available in Supplementary Materials.

Participants agreed that 19 welfare concerns were of high or medium priority for UK equids. These concerns can be summarised into the following areas: meeting equids’ ethological needs, use of equids in sport, equid management, breeding, transport, and veterinary care (Figure 3).

#### 3.3.2. Factors Contributing to Welfare Concerns

**Figure 4:**
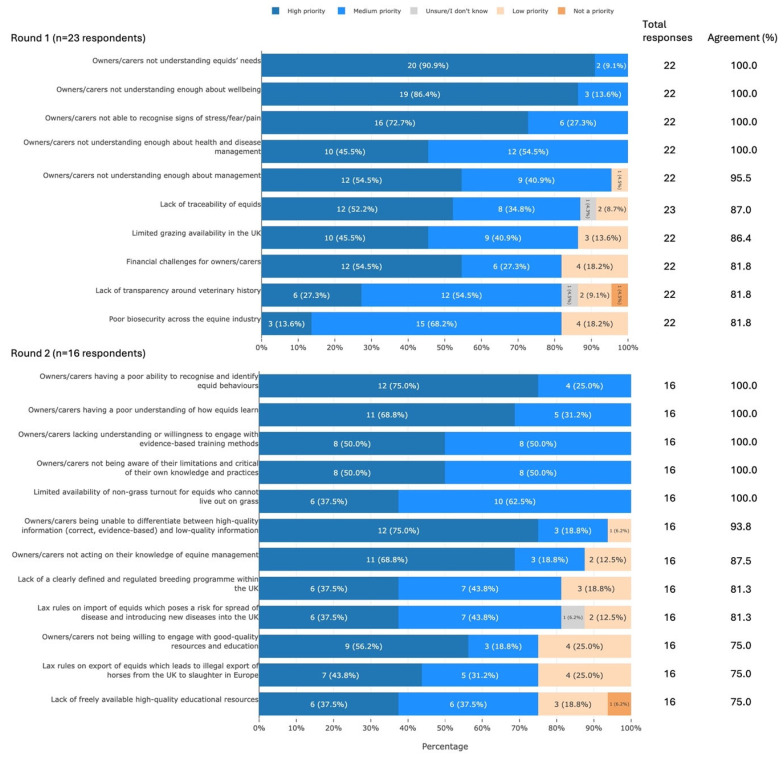
The 22 factors contributing to welfare concerns that met agreement, split by survey round and ranked by percentage agreement, from a Delphi study exploring stakeholders’ opinions about equid health and welfare and the related use of artificial intelligence. Within each statement, values may not sum to 100.0% due to rounding. Statements have been shortened for brevity. The full statements are available in Supplementary Materials. UK, United Kingdom.

Participants agreed that 22 factors contributing to welfare concerns in UK equids were of high or medium priority. These factors can be summarised into the following areas: level of owner/carer understanding, ability of owner/carer to recognise equid behaviour, overall monitoring of the equid population, human factors, availability of resources, and fundamental challenges (Figure 4).

#### 3.3.3. Areas for AI Development

**Figure 5:**
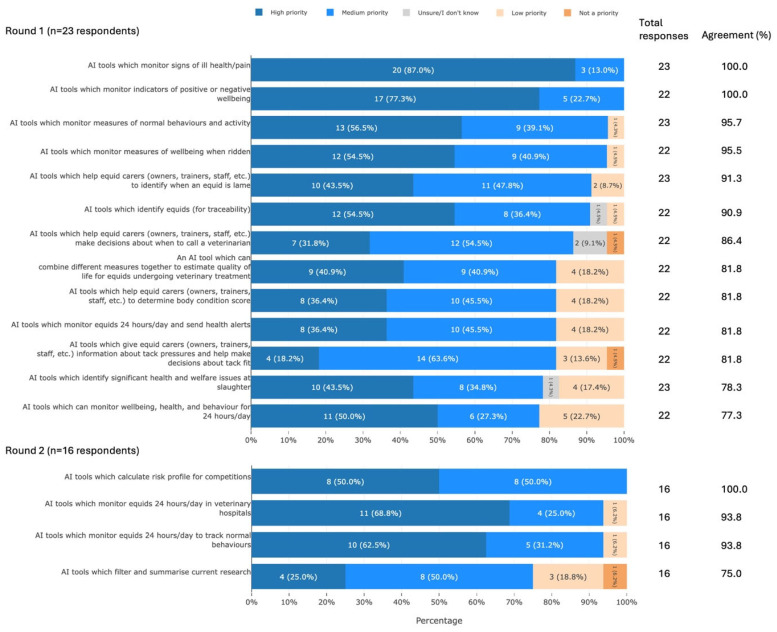
The 17 priority areas for AI development which met agreement, split by survey round and ranked by percentage agreement, from a Delphi study exploring stakeholders’ opinions about equid health and welfare and the related use of AI. Within each statement, values may not sum to 100.0% due to rounding. Statements have been shortened for brevity. The full statements are available in Supplementary Materials. AI, artificial intelligence.

Participants agreed that 17 areas for AI development were of medium or high priority for UK equid health and welfare. These areas can be summarised as assessment of equid wellbeing, individual equid monitoring, population-level monitoring, equid management, assessment of the ridden equid, and information analysis (Figure 5).

#### 3.3.4. Barriers Associated with the Development of AI Tools

**Figure 6:**
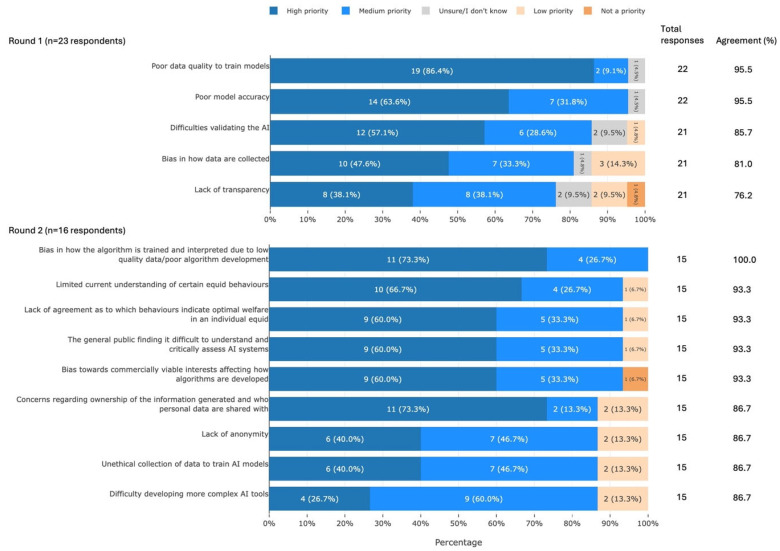
The 14 barriers associated with the development of AI tools that met agreement, split by survey round and ranked by percentage agreement, from a Delphi study exploring stakeholders’ opinions about equid health and welfare and the related use of AI. Within each statement, values may not sum to 100.0% due to rounding. Statements have been shortened for brevity. The full statements are available in Supplementary Materials. AI, artificial intelligence.

Participants agreed that 14 barriers associated with the development of AI tools were of high or medium priority for UK equid health and welfare. These barriers can be summarised into the following areas: data collection, model development, validation, ethics, limitations of the technology, and lack of public understanding (Figure 6).

#### 3.3.5. Barriers Associated with the Use of AI Tools

**Figure 7:**
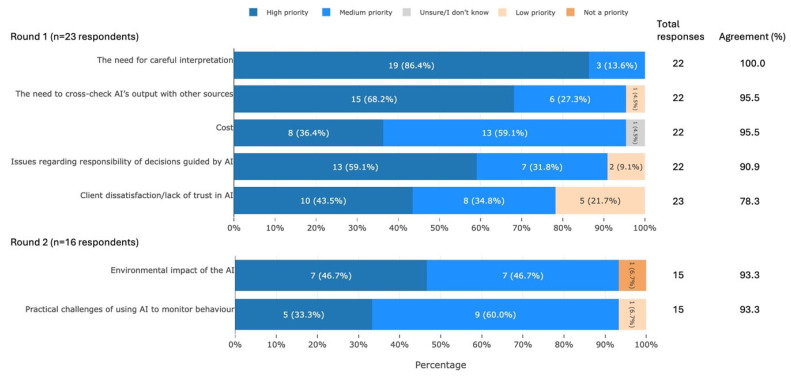
The seven barriers associated with the use of AI tools that met agreement, split by survey round and ranked by percentage agreement, from a Delphi study exploring stakeholders’ opinions about equid health and welfare and the related use of AI. Within each statement, values may not sum to 100.0% due to rounding. Statements have been shortened for brevity. The full statements are available in Supplementary Materials. AI, artificial intelligence.

Participants agreed that seven barriers associated with the use of AI tools were of high or medium priority for UK equid health and welfare. These barriers can be summarised into the following areas: the need for critical use, ethics, and fundamental considerations (Figure 7).

#### 3.3.6. Potential Problems Due to the Use of AI

**Figure 8:**
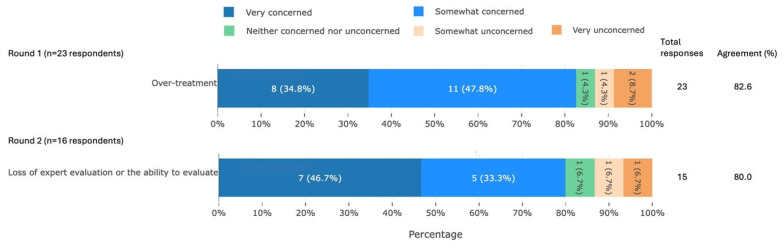
The two potential problems associated with the use of AI that met agreement, split by survey round, from a Delphi study exploring stakeholders’ opinions about equid health and welfare and the related use of AI. Within each statement, values may not sum to 100.0% due to rounding. Statements have been shortened for brevity. The full statements are available in Supplementary Materials. AI, artificial intelligence.

Participants agreed that two potential problems associated with the use of AI for UK equid health and welfare were somewhat or very concerning. These concerns relate to equid welfare and skill erosion in the equid industry (Figure 8).

#### 3.3.7. Solutions to Barriers/Problems

**Figure 9:**
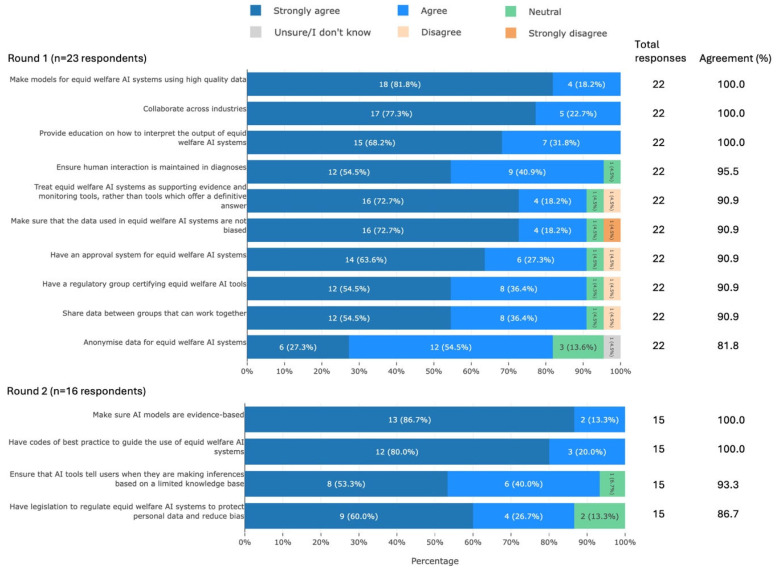
The 14 solutions to the barriers/problems associated with using AI that met agreement, split by survey round and ranked by percentage agreement, from a Delphi study exploring stakeholders’ opinions about equid health and welfare and the related use of AI. Within each statement, values may not sum to 100.0% due to rounding. Statements have been shortened for brevity. The full statements are available in Supplementary Materials. AI, artificial intelligence.

Participants agreed on 14 solutions to the barriers/problems associated with using AI for UK equid health and welfare. These solutions can be summarised into the following areas: development of evidence-based, unbiased AI systems, ethical development, careful use, education/guidance, approval/regulation, and collaboration (Figure 9).

#### 3.3.8. Ways to Make AI Tools Accessible and Useful

**Figure 10:**
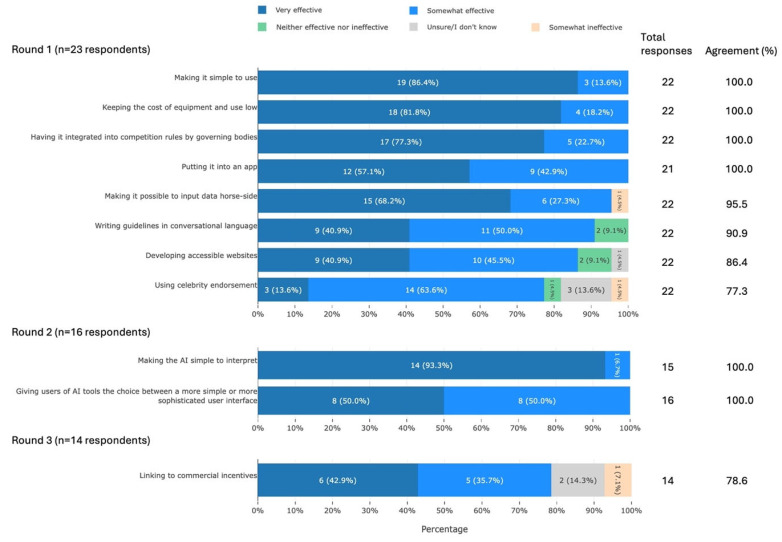
The 11 ways to make AI tools accessible and useful which met agreement, split by survey round and ranked by percentage agreement, from a Delphi study exploring stakeholders’ opinions about equid health and welfare and the related use of AI. Within each statement, values may not sum to 100.0% due to rounding. Statements have been shortened for brevity. The full statements are available in Supplementary Materials. AI, artificial intelligence.

Participants agreed on 11 ways that would be very or somewhat effective at making AI tools accessible and useful. These can be summarised into the following areas: user interface, regulation, financial considerations, and social factors (Figure 10).

## 4. Discussion

The aim of this study was to determine the main priorities for the development and use of AI tools to deliver the greatest benefits for equid health and welfare. We achieved this by recruiting stakeholders with a range of experiences and expertise, facilitating small group discussions to review current data and generate recommendations for future research and implementation, and reviewing these recommendations and generating a final consensus through an online Delphi survey.

Before discussing the results, we should summarise the study’s limitations. Firstly, the non-random selection of participants resulted in a cohort who were generally highly educated, ≥40 years old, and whose roles were skewed towards higher education, research, and regulation of equid welfare. The recruitment of AWRN members, in particular, may have contributed to possible bias, although only four participants were recruited through this route, and they were included within the welfare research stakeholder group. However, our results may not represent the opinions of younger people and groups such as coaches, riders, judges, and allied professionals (e.g., saddle fitters, equine physiotherapists, and equine nutritionists). Participants were predominantly female (78%) and this represents another potential bias. However, a female bias is typical of the horse owning/caring population, with many surveys describing over 90% female participants [17,18]. Another potential limitation is that opinions from the in-person workshop were anonymised. This was done to encourage participants to provide their honest opinions. It does, however, mean that participant age and background cannot be linked to individual suggestions or comments. Finally, our participants generally reported having a good understanding of AI and we specifically selected people who were involved in developing or testing AI systems. In summary, the participants’ demographics were predominantly female, had a higher level of higher education qualifications than the general UK or horse-owning population, and were likely to have a greater interest in welfare and AI than the general horse-owning population. Although probably not representative of the broader UK population, our cohort was well-placed to achieve the aim of a Delphi, which is to gather experts’ opinions on the topics under discussion: AI and equid welfare.

The high representation of welfare researchers among our participants may have influenced the resultant welfare priorities. There is, understandably, an association between an individual’s role in the industry and the welfare concerns they identify [19]. For example, individuals involved in charity work are likely to identify issues of high body condition score, overfeeding, and lack of water than those involved in ‘health’ or who are riders/trainers [19]. That said, other studies reporting the opinion of a wide range of stakeholders (including those involved in equine training and leisure use, and those with expertise in advocacy and community engagement) identified welfare issues that overlapped substantially with ours [12,20]. However, any summary of welfare concerns should be considered in conjunction with the demographics of the individuals involved in producing it.

Two of the authors (SF and RH) have involvement with a commercial AI company (outlined in the Conflicts of Interest Declaration). Stakeholders representing other AI companies also attended and contributed to the Delphi process. The workshop was structured to minimise any potential commercial biases arising from this. Participants were asked to identify welfare priorities in session 1, before listening to any presentations from the attending AI companies. Four companies gave presentations in session 2, and these provided examples of a range of different AI uses. The outcomes of the facilitated groups in the workshop were recorded by undergraduate students who had no commercial affiliations. The primary analysis of the workshop and Delphi outcomes was performed by PY, who was funded by AWRN and has no affiliations with a commercial AI company. The priorities, rankings, and recommendations were all based on quantitative analysis of participants’ anonymised suggestions and voting, ensuring that each stakeholder’s voice was represented.

Finally, there were potential biases within the study design. These included using a 4-point Likert scale for priorities and a 5-point scale for other rankings. The 4-point priority ranking (high, medium, low, none) was based on common descriptors but may have encouraged selection of the middle ranking or confused participants when other rankings were 5-point. There was also substantial participant drop out over the course of the Delphi process. This may mean that the agreement ratings in the final rounds were less representative of the original study cohort than those in the first round. However, our sample size is within the normal and acceptable range for Delphi studies [21,22].

### 4.1. Welfare Priorities

The priority welfare issues identified by the participants included failure to meet equids’ ethological needs (e.g., ‘friends, forage, and freedom to move’), suboptimal management (e.g., high worm burdens, obesity, insufficient veterinary care, stressful weaning methods), and riding and training issues (e.g., excessive rider weight, stressful training methods) (Figure 3). Several of these (the need for forage and social interaction, restrictive nosebands, and abrupt weaning) have previously been identified as having a severe adverse impact on welfare [20] and are echoed in other reports [12,19]. There are, however, some inconsistencies among studies. For example, our participants did not mention unstable social groups, whereas this has been reported previously [12]. While underfeeding was mentioned during the workshop, it was not identified as a priority issue. In contrast, hunger was identified as important in previous reports [12,19]. Overall, however, it appears that welfare priorities for UK equids have changed little in the past five, and perhaps even ten, years, indicating a need for more effective interventions.

### 4.2. Factors Contributing to Welfare Concerns

Participants were clear that factors relating to the individual owner/carer contributed to welfare concerns in UK equids, with many statements reaching 100% agreement. Many of these statements centred around owner/carer lack of knowledge/understanding (especially of equids’ needs, behaviour, and management) (Figure 4). This is consistent with previous work: 27/31 stakeholders (87%) in Horseman et al. [19] identified “lack of knowledge” as a source of welfare concerns, and themes of ignorance and lack of knowledge were identified in all three rounds of a Canadian Delphi study [23]. Studies aiming to directly assess owner/carer knowledge also support this view, with Watney et al. [24] suggesting that 1/3 of owners lack fundamental knowledge of equine management. The findings of the current study therefore contribute to a growing body of evidence indicating that a lack of knowledge of correct equid management may well be contributing to welfare concerns in the UK population. For this reason, owner/carer knowledge represents a target area for improvement.

Participants in the current study also agreed that owner/carer failure to understand equid behaviour and evidence-based training contributes to welfare concerns. This is consistent with previous work [12,19]. Other studies have identified specific areas of concern including recognition of pain [24], knowledge of learning theory [24], recognition of abnormal behaviour during tacking-up or mounting [25], and behaviour associated with negative affective states [26]. The current study highlights that improving owner/carer recognition of behaviour and understanding of training remains a target area for interventions aiming to improve UK equid welfare.

Participants in the current study highlighted several systemic factors contributing to welfare concerns in UK equids. This suggests that change is needed at more than the individual level. For example, poor traceability was a priority for 20/23 participants (87%). Despite the requirement in the UK for every equid to have a microchip, an up-to-date passport [27], and to be registered on the Central Equine Database (CED) [28], these conditions are often neglected, with a recent UK government report admitting that “much of the data on the CED is currently inaccurate and incomplete” [29]. Data relating to the fulfilment of equine identification requirements in the UK are mostly out of date, fragmented, and relate to subsets of the equid population only. For example, a recent report indicated that less than two thirds of Thoroughbreds’ passports are registered in the current owner’s name [30], and historical data indicate that only 30% of horses entering RSPCA care between 2015 and 2020 had been microchipped [31].

In the current study, the majority of participants (18/22; 82%) agreed that poor biosecurity contributed to welfare concerns. Arguably this was considered a lesser concern when compared to the previous work [12], where “lack of biosecurity and disease surveillance” was ranked as the most prevalent issue for the equine population. This difference may be due to the impacts of the 2019 equine influenza epidemic [32] being felt more strongly at that time. However, it is clear that infectious diseases continue to pose a risk to equid health and welfare [33], and that biosecurity in the UK equid industry is suboptimal [34]. Finally, the lack of a regulated breeding programme was identified as a welfare concern. Indiscriminate breeding is widely accepted as a contributor to the ‘horse crisis’ (the increasing number of horses at risk of neglect and abandonment which has placed substantial pressure on equine welfare charities) [31] and can be present even in sports such as Thoroughbred racing [35]. Looking more broadly at these systemic factors (poor traceability, lack of biosecurity, and unregulated breeding), it is evident that interventions are needed at both the individual and national level.

While the welfare issues and contributing factors identified in our study have been addressed in isolation above, they are often inter-linked. For example, Watney et al. [24] argued that an owner/carer who lacks knowledge is more likely to use inappropriate training techniques. This is supported by recent work showing that individuals who are less knowledge about learning theory tend to be more likely to use punishment in response to an unwanted equine behaviour compared to those with greater knowledge [36]. While this work only showed a statistically significant association between knowledge of learning theory and the likelihood of ‘shouting or using a whip to get the horse’s attention’, a non-significant trend was also found between knowledge of learning theory and the likelihood of ‘reprimanding the horse for disobeying’. In another example, a report by UK equine welfare charities raised concerns that delayed euthanasia may be a consequence of financial challenges [31]. Those in a position to influence welfare (e.g., welfare officers, veterinary practitioners, and policy makers) should therefore take a holistic approach to welfare issues, identifying and targeting the root causes where possible.

### 4.3. Areas for AI Development

The priority areas for AI development identified by participants in the current study showed a strong focus on tools that would assist with assessment of equid behaviour, activity, and mental and physical wellbeing, including when ridden (Figure 5). Tools based on AI have either already been developed or have shown proof of concept in many of these areas. These include monitoring illness (detection of lameness [37,38], equine exercise-induced pulmonary haemorrhage (EIPH) [39], and induced colic [40]) and overall wellbeing (detection of pain [41,42] and fatigue [43], as well as assessment of comfort in ridden horses [44]). Detection of behaviour is a popular area for the use of AI and it is therefore unsurprising that a multitude of tools have been developed to detect equid behaviour [45,46,47,48]. AI tools can also identify individual horses [49,50] and tools to calculate risk profile may be feasible. While the accuracy of these tools varies, it seems that the AI industry is already responding to the need—confirmed by the current study—for welfare-relevant tools.

However, development of more specific or integrative tools is slow. For example, the authors are not aware of an AI tool to assess tack pressure and fit, determine equine body condition score (BCS), provide assistance on when to call a veterinarian, or estimate quality of life (QoL)—all areas identified as a priority by our participants. Potential reasons for this include lack of demand, financial unviability, and difficulties in development. For example, despite the value offered by a QoL assessment tool, there are no validated QoL assessment tools for horses [51]. This may be due to the challenges associated with aggregating multiple measures into one score [52]. These difficulties would need to be addressed before an AI tool could be developed.

Many of the AI tools identified for development represent potential solutions to the welfare concerns identified by participants. While such tools are only one part of the puzzle (a broad suite of interventions is likely to be most effective at improving equid welfare), they may play an important role. Tools to monitor illness, wellbeing, and behaviour may help to address the priority welfare issues of ethological needs not being met, social isolation, confinement, and poor welfare during travel. Tools to identify lameness, when to call a veterinarian, and sending health alerts may help to address insufficient veterinary care. A tool to identify equids could help to address the lack of traceability and lack of transparency around veterinary histories, and a tool to give information on tack pressure and fit could help to address issues with tack fit. The AI industry should therefore focus on developing tools in areas that were identified as priorities for development and are clearly linked to priority welfare concerns.

### 4.4. Barriers Associated with the Development of AI Tools

Participants identified a wide range of barriers to the development and use of AI tools for equid health and welfare. Key among these were the use of biased or ‘poor-quality’ data to train AI models, as well as issues with model validation and accuracy (Figure 6). Many of these barriers are fundamental to the development of AI tools. Data quality significantly affects the reliability of an AI tool [53], and its importance is recognised in tools for animal and human health [54,55,56]. Bias in the dataset is a well-documented issue [57,58,59,60,61], which limits the applicability of the model to a broader population [62]. This can also contribute to poor accuracy, particularly when the AI tool is applied to a new context [63]. Accuracy can also vary due to the type of model used and the context in which it is being applied [64].

Other barriers identified by our participants also echo wider concerns. For example, transparency is the most commonly mentioned principle in global AI guidelines [65] and is considered important in veterinary medicine [66]. There is also clear consensus on the need to consider ownership and use of personal data [55,57,59,66,67]. The recognition that animals cannot provide informed consent [55] may contribute to concerns about unethical collection of data, and development of equid AI tools should therefore ensure that data collection is ethical. Finally, it is broadly accepted that AI systems are fundamentally hard to interpret, especially those developed using deep learning models [63] and ‘blackbox’ AI [66]. It is therefore unsurprising that the public can find it difficult to critically assess these tools.

Taken together, it is clear that many of the participants’ concerns are already well-established priorities for AI development in other spheres. This indicates that the development of AI tools for equid health and welfare should follow general guidelines and best practices. However, participants also highlighted some barriers that may be uniquely applicable to the development of animal- and equid-specific AI tools. For example, the limited consistency of, and lack of objective evidence to support, equid behavioural identification [68] likely contributes in part to a lack of agreement over the welfare implications of some equid behaviours. The recognition that individual equids differ in their expression of an underlying emotion [26] further complicates interpretation of behaviour. More complex AI tools may be particularly challenging to develop in animals due to difficulties interpreting behaviour [69] and environmental variability [70]. Taken together, these findings suggest that the industry should prioritise fundamental understanding of equid behaviour in order to enable validation of behavioural AI tools.

### 4.5. Barriers Associated with the Use of AI Tools

The key barriers raised by participants in relation to the use of equid AI tools included the need for care in the interpretation and use of AI outputs, cost, and environmental impact (Figure 7). These also represent issues associated with the use of AI more generally. Users tend to interpret AI outputs as representing reality [58] rather than considering the limitations of the model—something which Long & Magerko [71] identified as an important skill in ‘AI literacy’. The need to increase AI literacy in end-users is likely to become more urgent as AI tools become increasingly available and accessible to horse owners/carers [5]. The growth of AI has also raised concerns about sustainability [59,66,72,73] and the involvement of AI in responsible decision-making [56,59,66,74,75], issues that are common to the fields of human and animal health. Equid AI tools should therefore be developed in accordance with general guidance, some of which already exists [76].

The fundamental barriers raised by participants (cost and practical challenges) are commonly mentioned in animal-related AI applications. Cost may decrease in the future as the industry develops [77], for example, through mass production of device components [78], but it is currently mentioned as a key challenge to the use of AI in veterinary medicine [55,74] and in monitoring equine behaviour [70]. The practical challenges of using AI to monitor equines are also recognised [70]. Solutions to these challenges may be found by considering AI use in other fields.

### 4.6. Potential Problems Due to the Use of AI

The majority of participants (19/23, 83%) were concerned about use of AI resulting in over-treatment (Figure 8). AI has been suggested to contribute to misdiagnosis [67] and overdiagnosis [66], and over-reliance on AI may further exacerbate the issue [79]. However, AI tools have been shown to reduce over-treatment in human medicine by reducing defensive medicine and lack of knowledge [80]. This may explain the mixed results of our participants, with two participants being ‘unconcerned’. AI tools that offer clinical guidance should therefore be developed with the aim of reducing misdiagnosis and over-treatment by the user.

Most participants (12/15, 80%) were concerned that the use of AI would result in loss of evaluation skills in the user. There is evidence of a negative correlation between use of AI tools and critical thinking [81], and it has been suggested that over-reliance on intelligent decision support tools may erode veterinarians’ skills [66]. The development of an AI tool that can reliably diagnose 40 common equine diseases [82] highlights the immediacy of this concern in the veterinary field. However, this loss of evaluation may be mitigated by expertise and experience. In a simulation study, Fan et al. [83] showed that experts were much less likely to accept incorrect answers from an automated agent than novices. If the same holds true for AI tools, this suggests that expertise may allow individuals to retain the ability to evaluate. Encouraging individuals to develop expertise in their field is therefore important amid an increasing abundance of AI tools.

### 4.7. Solutions to Barriers/Problems

Many of the solutions that participants identified as helping AI to effectively improve welfare—‘high-quality’ data, lack of bias, AI’s acknowledgement of its limitations, anonymity and data privacy, and human involvement (Figure 9)—are captured in best practice guidelines such as the “Ethics guidelines for trustworthy AI” [76]. More specific guidance also exists for each proposed solution. To reduce bias, for example, developers should use a diverse, representative training dataset [74,84], add metadata to the dataset specifying how the data were collected and annotated, and consider using a de-biasing tool [84]. Guidance is also available on quantifying and reducing the risk of de-anonymisation of the dataset [85]. This indicates that the development of AI tools for equid health and welfare should follow standard best practice guidelines.

Some solutions identified by participants go beyond general guidance and so warrant further discussion. All participants agreed that providing education on how to interpret AI systems would be helpful. Education on AI contributes to public understanding of the technology [71], has been shown to increase learners’ self-reported AI literacy and competence [86], and has even been recommended as a way to prevent malicious use of AI by developers [87]. This demonstrates the power of education to improve AI literacy and potentially mitigate the problems that may result from users’ misplaced trust in AI outputs. To facilitate this, education should be made widely available and AI users should be encouraged to engage with it.

Participants also expressed the need for approval and regulation of AI systems, a sentiment that is echoed across the charity and veterinary sectors [55,57,88]. There is currently no general regulation of AI in the UK [8], leaving a large gap between ethical guidelines and the law [89] and highlighting the need to move from “principles to processes” for AI regulation [90]. There is evidence of such a move in the European Union, as evidenced by the AI Act [91], but UK approval and regulation of AI tools for equid health is lagging, despite demand.

Finally, participants highlighted collaboration as a priority. One key way in which collaboration can be useful for AI development is through data-sharing [92]. This can help to build diverse, representative training datasets but comes with challenges. However, application of the FAIR principles [93,94], use of standardised data-sharing agreements [55], and innovative data privacy techniques [92] have made such sharing feasible. As such, the industry should welcome collaboration and data-sharing in order to generate more reliable AI systems.

### 4.8. Ways to Make AI Tools Accessible and Useful

Many of the strategies that participants thought would be effective at making AI tools accessible and useful focused on tools’ complexity and ease-of-use. Complexity is considered to be one of the key factors determining adoption of a new innovation [95,96], and perceived ease-of-use was shown to be relevant in the adoption of essential technology on farms [97]. One innovative suggestion was to allow users to select the complexity of their interface. This echoes a similar suggestion by Ribera & Lapedriza [98]: to match the AI’s explanation to the user’s level of AI expertise. Overall, any strategies that make the tool simpler and more user-friendly are likely to increase accessibility and uptake.

## 5. Conclusions

The field of AI is growing rapidly, and AI systems are already being used for a variety of applications in veterinary medicine and the equid industry. This study has identified key areas of equid health and welfare where AI is likely to have the greatest benefit, and these should be used to inform the direction of future funding and industry development. The concerns about the development and use of AI for equids raised by the stakeholders in this study are similar to those raised for AI in general. Regulations and guidance developed for AI tools generally could therefore also be used to guide the development of AI for equid health and welfare. The key areas of priority, main concerns and potential barriers, and solutions proposed for the way forward outlined in this study represent the first part of a framework for the use of AI in equid health and welfare.

## Figures and Tables

**Table 1:** Aims, introductory talks and presentations, and discussion points for three sessions in a workshop exploring stakeholders’ opinions about equid health and welfare and the related use of artificial intelligence (AI).

Session Number	Aim	Introductory Talk and Presentations	Discussion Points
Session 1	To identify welfare priorities in the UK equid population	Talk: “What is welfare?”—an introduction to equid welfare and its importance	What are the current welfare priorities for the equid population in the UK? Initial open discussion extended by reviewing welfare priorities published in Rioja-Lang et al. [12]
Session 2	To identify how AI can and should be used to improve equid welfare	Talk: “What is AI?”—an introduction to AI Short presentations from a range of companies already using AI and computer technologies to improve equid health, welfare, and monitoring	How can AI be used most effectively to drive welfare improvements? How can the equid community work together to drive welfare change? Two main themes were explored: ○ Data access and information-sharing ○ How organisations and industries can work together
Session 3	To identify challenges and concerns about using AI to improve equid welfare	Talk: “Known challenges to the development of AI technologies” Presentation of new data on equid owners’ perspectives on the use of AI	What are the potential problems with, and barriers to, using AI to improve equid welfare? How can we solve these?

Abbreviations: AI, artificial intelligence; UK, United Kingdom.

**Table 2:** Number of participants by stakeholder group at a workshop that explored stakeholders’ opinions about equid health and welfare and the related use of AI.

Stakeholder Group	Target Number of Participants	Actual Number (%) ^†^ of Participants
Veterinarian	10	10 (24.4%)
Equid owner/carer/coach	10	6 (14.6%)
Welfare researcher	5	7 (17.1%)
Welfare adviser	10	5 (12.2%)
Regulatory body	5	5 (12.2%)
AI company	5	4 (9.8%)
AWRN member	5	4 (9.8%)

^†^ Data may not sum to 100.0% due to rounding; Abbreviations: AI, artificial intelligence; AWRN, Animal Welfare Research Network.

**Table 3:** Themes and sub-themes identified during the workshop that explored stakeholders’ opinions about equid health and welfare and the related use of AI.

Theme	Sub-Themes
(1) Current welfare concerns	(1a) Welfare priorities (1b) Factors contributing to welfare concerns
(2) Areas for AI development	N/A (no sub-themes identified)
(3) Barriers/problems associated with AI	(3a) Barriers associated with the development of AI tools (3b) Barriers associated with the use of AI tools (3c) Potential problems due to use of AI tools
(4) Solutions to barriers/problems	(4a) Solutions to barriers/problems (4b) Ways to make AI tools accessible and useful

Abbreviations: AI, artificial intelligence; N/A, not applicable.

**Table 4:** Summary of the demographic data collected from the participants in the first-round survey from a Delphi study exploring stakeholders’ opinions about equid health and welfare and the related use of AI.

Variable	Category	Number (%) ^†^
Gender	Female	18 (78.3%)
	Male	5 (21.7%)
Age (years)	20–29	2 (8.7%)
	30–39	4 (17.4%)
	40–49	8 (34.8%)
	50–59	7 (30.4%)
	60+	2 (8.7%)
Highest level of education completed	Further education (post-secondary)	1 (4.3%)
	Higher education (university level)	5 (21.7%)
	Masters’ degree	4 (17.4%)
	Veterinary degree	1 (4.3%)
	PhD	12 (52.2%)
Involvement in equid health and welfare ^‡^	“I own/care for equid(s)”	18 (78.2%)
	“I compete in equestrian competitions”	9 (39.1%)
	“I judge, officiate, or steward at equestrian competitions”	5 (21.7%)
	“I teach or train equids and/or equid owners or riders”	3 (13.0%)
	“I am an allied equine professional (e.g., saddle fitter, equine physiotherapist, equine nutritionist)”	1 (4.3%)
	“I am part of a clinical equine veterinary team”	8 (34.8%)
	“I deliver public education about equid health and welfare”	14 (60.9%)
	“I deliver higher education about equid health and welfare”	13 (56.5%)
	“I undertake research about equid health and welfare”	12 (52.2%)
	“I develop or deliver resources which support equid health and welfare”	9 (39.1%)
	“I develop or distribute industry guidance or regulations for equid health and welfare”	11 (47.8%)
Current understanding of artificial intelligence (AI)	“Heard of it but not sure what it was before the workshop”	1 (4.3%)
	“Could understand a conversation on it”	10 (43.5%)
	“Could take part in a conversation on it”	9 (39.1%)
	“Involved in making or testing AI systems”	3 (13.0%)

^†^ Within variable, data may not sum to 100.0% due to rounding. ^‡^ Statements shortened for brevity. The full statements are available in Supplementary Materials. Abbreviations: AI, artificial intelligence.

## Data Availability

The free-text comments were given under the condition of anonymity and are thus not available for sharing. The rest of the data generated and analysed during the current study are available from the corresponding author on reasonable request.

## Supplementary Materials

The following supporting information can be downloaded at https://www.mdpi.com/article/10.3390/ani16071082/s1, File S1: Surveys sent to participants.
