# Supplementary material for: Priorities and Recommendations for Using Artificial Intelligence (AI) to Improve Equid Health and Welfare
Source: Animals (Basel). 2026 Apr 1;16(7):1082. doi: 10.3390/ani16071082 (PMC13072182; doi:10.3390/ani16071082)
Supplement: Supplementary file 1 [file animals-16-01082-s001.zip › animals-4189183-supplementary.pdf]

Questionnaire round 1: Use of artificial intelligence to improve equid health and welfare

Thank you for your contributions at the workshop at The School of Veterinary Medicine and Science, University of Nottingham on Wednesday 29th January 2025.
Since the workshop, we have been reviewing all the fantastic suggestions and ideas, and categorising and grouping them together. We would now really appreciate your help in identifying the most important priorities going forwards. This questionnaire shares the summarised results from the workshop and asks you to rank, prioritise or indicate your agreement on them.

When you submit this form, it will not automatically collect your details like name and email address unless you provide it yourself.

* Required

Definition of terms, consent form, and further contact

Key terms – Please note that throughout this survey we use the term "equid" to include horses, ponies, donkeys, and their hybrids.

Consent form – You must complete this form in order to complete this survey.

Participation in this research is entirely voluntary and there is no obligation to take part. Please note you must be over 18 years of age to participate. This study has been approved by the University of Nottingham, School of Veterinary Medicine and Science Ethics Committee. Further information can be obtained by contacting the research team at sarah.freeman@nottingham.ac.uk.

As a participant in this study you can:

- Request to see a copy/summary of the completed study
- Request to see any information written down/kept during the process of data collection

1. This consent form is a formal way of indicating that you agree to participate in this study. Please read the statements below and tick the boxes to show you agree: *

☐ I understand that I am not obliged to give consent and I may withdraw my participation at any point of the process

☐ I understand that my contribution to the study will be recorded and used for research purposes

☐ I understand that the research from this study may be presented at research conferences or meetings

2. Please can you provide your email if you are happy for us to contact you about any feedback you give on the survey (e.g., for further clarification of points you have raised).

Enter your answer

Demographics and relevant experience

These first few questions are about you. They will help us provide an overview of the people attending and contributing to the workshop outcomes.

3. Which age category do you fit into?

- ◯ 18–19 years
- ◯ 20–29 years
- ◯ 30–39 years
- ◯ 40–49 years
- ◯ 50–59 years
- ◯ 60+ years

4. How would you describe your gender?

- ◯ Male
- ◯ Female
- ◯ Non-binary
- ◯ Other

5. What is the highest level of education you have completed?

- ◯ Secondary school (e.g. GCSEs, Level 2 qualification)
- ◯ Further education (e.g. A-levels, Level 3 qualification)
- ◯ Higher education (e.g. Bachelor's, Level 4-6 qualification)
- ◯ Masters' degree
- ◯ PhD
- ◯ Prefer not to say
- ◯ Other

6. Which of the following describe your involvement in equid health and welfare (please tick all that apply)?

- ☐ I own/care for equid(s) (including owning or loaning equids, or managing a yard)
- ☐ I compete in equestrian competitions
- ☐ I judge, officiate, or steward at equestrian competitions
- ☐ I teach or train equids and/or equid owners or riders
- ☐ I am an allied equine professional (e.g. saddle fitter, equine physiotherapist, equine nutritionist)
- ☐ I am part of a clinical equine veterinary team (e.g. veterinarian or nurse providing clinical care to equids)
- ☐ I deliver public education about equid health and welfare (including talks, campaigns, and one-to-one support for owners)
- ☐ I deliver higher education about equid health and welfare (including teaching on College or University courses)
- ☐ I undertake research about equid health and welfare (e.g., as a postgraduate student or senior researcher)
- ☐ I develop or deliver resources which support equid health and welfare (e.g., software programmes, practical support)

- [ ] I develop or distribute industry guidance or regulations for equid health and welfare (e.g., regulations on use of the whip, guidance on infectious disease control)
- [ ] Other

7. Which would you say best fits your current understanding of artificial intelligence (AI)?

- ○ Heard of it but not sure what is was before the workshop
- ○ Could understand a conversation on it
- ○ Could take part in a conversation on it
- ○ Involved in making or testing AI systems

What are the current priorities for UK equid welfare?

8. The workshop participants identified the following areas as current welfare concerns for UK equids. Please select how you think we should prioritise each of the suggested areas in order to improve UK equid welfare.

Guidance: In deciding the priority level, consider the total number of equids affected (i.e., the prevalence of the welfare concern), the impact it has on the welfare of individual affected equids (i.e., its severity), and how long that negative impact typically lasts (i.e., its duration).

	High priority	Medium priority	Low priority	Not a priority	Unsure/I don't know
Obesity	○	○	○	○	○
Inappropriate use of supplements (e.g., giving too many or not providing enough to meet the equid's needs)	○	○	○	○	○
Underfeeding	○	○	○	○	○
High worm burdens	○	○	○	○	○
Basic needs (friends, forage, freedom to move) not being met	○	○	○	○	○
Weaning methods which cause stress/distress	○	○	○	○	○

Delayed euthanasia (defined as when an animal no longer has a good quality of life, and not euthanising sooner has resulted in prolonged and unnecessary suffering)	○	○	○	○	○
Insufficient veterinary care (e.g., veterinary care is delayed or not sought)	○	○	○	○	○
Excessive veterinary intervention (e.g., invasive procedures that are not necessary and over-medication – including excessive use of joint medication)	○	○	○	○	○
Confinement (e.g., equids in an enclosed space for a long time without areas to run freely)	○	○	○	○	○
Social isolation (e.g., equids not being able to see and/or touch other equids)	○	○	○	○	○
Poor welfare during travel (e.g., long or frequent periods of travel, stress during travel)	○	○	○	○	○
Riders being too heavy for their horses	○	○	○	○	○

Incorrect matching of horses and riders (e.g., inexperienced riders with reactive horses)	○	○	○	○	○
Tack issues (e.g., poorly fitted tack causing pain or stress/distress, over-tacking)	○	○	○	○	○
Injuries from sport (e.g., tendon issues) sustained in training or competition	○	○	○	○	○
Training approaches which stress/distress the equid or don’t adequately prepare them for their career	○	○	○	○	○
Poor wellbeing of sport horses after their career (e.g., not rehomed appropriately, or kept in environments which don’t meet their needs)	○	○	○	○	○
Equids being bred with poor conformation or inherited health issues	○	○	○	○	○
Equids being bred with temperaments only suitable for limited purposes, which make them difficult to manage for the majority of equid owners	○	○	○	○	○

9. The workshop participants identified the following areas as factors contributing to current welfare concerns for UK equids. Please select how you think we should prioritise each of the suggested areas in order to improve UK equid welfare.

Guidance: In deciding the priority level, consider the total number of equids affected (i.e., the prevalence of the welfare concern), the impact it has on the welfare of individual affected equids (i.e., its severity), and how long that negative impact typically lasts (i.e., its duration).

	High priority	Medium priority	Low priority	Not a priority	Unsure/I don't know
Owners/carers not understanding enough about health and disease management (e.g., body condition scoring, disease prevention and control)	○	○	○	○	○
Owners/carers not understanding enough about management (e.g., land management, appropriate housing of equids, use of appropriate tack)	○	○	○	○	○
Owners/carers not understanding enough about wellbeing (e.g., welfare and quality of life, building equine social groups)	○	○	○	○	○
Owners/carers not understanding equids' needs (e.g., the need for forage, friends, and freedom to move)	○	○	○	○	○
Owners/carers not able to recognise signs of stress/fear/pain	○	○	○	○	○

Limited access to good-quality information within the equine industry	○	○	○	○	○
Limited availability of education for owners/carers of equids	○	○	○	○	○
Lack of traceability of equids (e.g., inability to track a horse through its lifetime)	○	○	○	○	○
Lack of transparency around veterinary history (e.g., not sharing previous veterinary history)	○	○	○	○	○
Lax rules on import/export of equids	○	○	○	○	○
Lack of control of breeding	○	○	○	○	○
Financial challenges for owners/carers (e.g., insufficient funds for full veterinary care, insufficient land for turnout)	○	○	○	○	○
Limited grazing availability in the UK (e.g., amount of grazing available across the UK or on some livery yards)	○	○	○	○	○
Overbreeding of equids	○	○	○	○	○
Humanising of equids by owners/carers	○	○	○	○	○

Poor biosecurity across the equine industry (e.g., insufficient strangles testing and alerting)	○	○	○	○	○

10. Are there any comments you would like to make on this section about current welfare concerns for UK equids?

Enter your answer

How can we use artificial intelligence to solve the current welfare issues?

11. The workshop participants recommended several areas where we should be developing artificial intelligence (AI) to improve equid health and welfare. These are listed below. Please select how you think we should prioritise each of the suggested areas for AI development.

	High priority	Medium priority	Low priority	Not a priority	Unsure/I don't know
AI tools which monitor measures of normal behaviours and activity (e.g., temperature, eating, sleeping, and social interactions)	○	○	○	○	○
AI tools which monitor indicators of positive or negative wellbeing (e.g., by identifying behaviours and mental state)	○	○	○	○	○
AI tools which monitor signs of ill health/pain (e.g., lameness, facial expressions of pain)	○	○	○	○	○
AI tools which monitor measures of wellbeing when ridden (e.g., measures of tack fit and equipment use, facial indicators of wellbeing)	○	○	○	○	○

An AI tool which can combine different measures together to estimate quality of life for equids undergoing veterinary treatment	○	○	○	○	○
AI tools to help competition judges to rank entrants	○	○	○	○	○
AI tools which help equid carers (owners, trainers, staff, etc.) make decisions about whether an equid needs a rug	○	○	○	○	○
AI tools which help equid carers (owners, trainers, staff, etc.) make decisions about when to call a veterinarian	○	○	○	○	○
AI tools which help equid carers (owners, trainers, staff, etc.) make decisions about wound care on the yard	○	○	○	○	○
AI tools which help equid carers and health professionals make decisions about injury and rehabilitation plans	○	○	○	○	○

AI tools which give equid carers (owners, trainers, staff, etc.) information about tack pressures and help make decisions about tack fit	○	○	○	○	○

12. [Continued from the previous question] The workshop participants recommended several areas where we should be developing artificial intelligence (AI) to improve equid health and welfare. These are listed below. Please select how you think we should prioritise each of the suggested areas for AI development.

	High priority	Medium priority	Low priority	Not a priority	Unsure/I don't know
AI tools which help equid carers (owners, trainers, staff, etc.) to determine body condition score	○	○	○	○	○
AI tools which help equid carers (owners, trainers, staff, etc.) to identify when an equid is lame	○	○	○	○	○
AI tools which digitise procedures (e.g., faecal worm egg counting [FWEC], semen analysis)	○	○	○	○	○
AI tools which track and predict performance	○	○	○	○	○
AI tools which calculate risk profile for competitions	○	○	○	○	○
AI tools which identify equids (for traceability)	○	○	○	○	○

AI tools which identify significant health and welfare issues at slaughter	◯	◯	◯	◯	◯
AI tools which filter and summarise current research	◯	◯	◯	◯	◯
AI tools which can monitor wellbeing, health, and behaviour for 24 hours/day	◯	◯	◯	◯	◯
AI tools which monitor equids 24 hours/day in veterinary hospitals	◯	◯	◯	◯	◯
AI tools which monitor equids 24 hours/day and send health alerts	◯	◯	◯	◯	◯
AI tools which monitor equids 24 hours/day to track normal behaviours	◯	◯	◯	◯	◯

13. The workshop participants suggested several ideas of how we can make AI tools accessible and useful. Please select how effective you think each suggestion would be for making a robust AI tool accessible and useful for horse owners/carers.

	Very effective	Somewhat effective	Neither effective nor ineffective	Somewhat ineffective	Very ineffective	Unsure/I don't know
Using celebrity endorsement	◯	◯	◯	◯	◯	◯
Writing guidelines in conversational language	◯	◯	◯	◯	◯	◯

Developing accessible websites	◯	◯	◯	◯	◯	◯
Putting it into an app	◯	◯	◯	◯	◯	◯
Making it simple to use	◯	◯	◯	◯	◯	◯
Making it possible to input data horse-side	◯	◯	◯	◯	◯	◯
Making it into a competition (i.e., users can see how their horse ranks against others)	◯	◯	◯	◯	◯	◯
Making it into a game, offering people rewards	◯	◯	◯	◯	◯	◯
Keeping the cost of equipment and use low	◯	◯	◯	◯	◯	◯
Having it integrated into competition rules by governing bodies (e.g., automating the checking of vaccination records)	◯	◯	◯	◯	◯	◯

14. Are there any comments you would like to make on this section about how we could use AI to solve welfare issues?

Enter your answer

What are the potential problems and barriers to using AI to improve equid welfare?

15. The workshop participants identified potential problems which could arise from using AI in equid welfare. Please select how concerned you are about each of these potential problems that were identified.

	Very concerned	Somewhat concerned	Neither concerned nor unconcerned	Somewhat unconcerned	Very unconcerned	Unsure/I don't know
Over-treatment (unnecessary or excessive medical interventions that do not benefit the equid or where the risks outweigh the potential benefits)	◯	◯	◯	◯	◯	◯
Under-treatment (insufficient or inadequate treatment, which fails to provide the necessary care to the equid)	◯	◯	◯	◯	◯	◯
Skill erosion of veterinarians	◯	◯	◯	◯	◯	◯
Loss of human influence in decision-making	◯	◯	◯	◯	◯	◯
Reduced human curiosity	◯	◯	◯	◯	◯	◯

16. The workshop participants identified several potential barriers to using AI to improve equid welfare. These are split into two categories: barriers relating to the <u>development</u>, and barriers relating to the <u>use</u> of AI tools. For each category, please select how you think we should prioritise solving each of these barriers in order to improve equid welfare.

These barriers relate to the development of AI tools. Please select how you think we should prioritise solving each of these barriers in order to improve equid welfare.

	High priority	Medium priority	Low priority	Not a priority	Unsure/I don't know
Poor data quality to train models	◯	◯	◯	◯	◯
Poor model accuracy	◯	◯	◯	◯	◯
Bias towards commercially viable interests	◯	◯	◯	◯	◯
Bias in how data are collected	◯	◯	◯	◯	◯
Bias in how the algorithm is trained and interpreted	◯	◯	◯	◯	◯
Concerns regarding ownership of the information generated	◯	◯	◯	◯	◯
The general public finding it difficult to understand and critically assess AI systems (e.g., how is AI using the data)	◯	◯	◯	◯	◯
Lack of anonymity	◯	◯	◯	◯	◯
Difficulties validating the AI	◯	◯	◯	◯	◯
Lack of transparency	◯	◯	◯	◯	◯

17. These barriers relate to the use of AI tools. Please select how you think we should prioritise solving each of these barriers in order to improve equid welfare.

	High priority	Medium priority	Low priority	Not a priority	Unsure/I don't know
The need for careful interpretation	◯	◯	◯	◯	◯
The need to cross-check AI's output with other sources	◯	◯	◯	◯	◯
Cost	◯	◯	◯	◯	◯
Issues regarding responsibility of decisions guided by AI	◯	◯	◯	◯	◯
Client dissatisfaction/lack of trust in AI	◯	◯	◯	◯	◯
Environmental impact of the AI	◯	◯	◯	◯	◯

18. Are there any comments you would like to make on this section about the potential barriers to, and problems with, using AI to solve welfare concerns?

Enter your answer

How can we solve these problems?

19. The workshop participants proposed solutions to how we can help AI to effectively improve equid welfare. These are listed below. Please select whether you agree, disagree or are neutral for each of the statements below relating to suggested solutions.

	Strongly agree	Agree	Neutral	Disagree	Strongly disagree	Unsure/I don't know
Have an approval system for equid welfare AI systems	○	○	○	○	○	○
Have legislation to regulate equid welfare AI systems	○	○	○	○	○	○
Share data between groups that can work together (e.g., AI companies)	○	○	○	○	○	○
Anonymise data for equid welfare AI systems	○	○	○	○	○	○
Make sure that the data used in equid welfare AI systems are not biased	○	○	○	○	○	○
Make models for equid welfare AI systems using high quality data	○	○	○	○	○	○

Treat equid welfare AI systems as supporting evidence and monitoring tools, rather than tools which offer a definitive answer	○	○	○	○	○	○
Provide education on how to interpret the output of equid welfare AI systems	○	○	○	○	○	○
Ensure human interaction is maintained in diagnoses (e.g., A human is always involved in the diagnosis provided)	○	○	○	○	○	○
Focus on welfare/management tools rather than short-term diagnostics	○	○	○	○	○	○
Have a regulatory group certifying equid welfare AI tools	○	○	○	○	○	○
Collaborate across industries	○	○	○	○	○	○

20. Are there any comments you would like to make on this section about the potential solutions to the problems of using AI for equid health and welfare?

Enter your answer

Questionnaire round 2: Use of artificial intelligence to improve equid health and welfare

Thank you for taking the time to complete round 1 of this questionnaire in May. Your generosity is very much appreciated and you have already identified clear priorities for the use of AI for equid welfare, which is fantastic.

We have now worked through the results and, for each question, have grouped the statements (welfare concerns/potential uses of AI/concerns about AI) into three categories:

- Important (>75% of people thought this was important)
- Unsure (25-75% of people thought this was important)
- Do not agree (<25% of people thought this was important)

We then removed the statements categorised as 'Important' or 'Do not agree' and changed the wording of the statements categorised as 'Unsure' to make them clearer. We also added in any new ideas that participants gave in the free text boxes.

We would now really appreciate your input to help us to identify which of these updated/new statements are important priorities going forward. As previously, this questionnaire asks you to prioritise or indicate your agreement on a variety of statements.

When you submit this form, it will not automatically collect your details like name and email address unless you provide it yourself.

* Required

Definition of terms, consent form, and further contact

Key terms – Please note that throughout this survey we use the term "equid" to include horses, ponies, donkeys, and their hybrids.

Consent form – You must complete this form in order to complete this survey.

Participation in this research is entirely voluntary and there is no obligation to take part. Please note you must be over 18 years of age to participate. This study has been approved by the University of Nottingham, School of Veterinary Medicine and Science Ethics Committee. Further information can be obtained by contacting the research team at sarah.freeman@nottingham.ac.uk.

As a participant in this study you can:

- Request to see a copy/summary of the completed study
- Request to see any information written down/kept during the process of data collection

1. Did you complete round 1 of this questionnaire (titled "Use of AI to improve equid health and welfare", available between May 1st and May 23rd)? *

   Due to our methodology we are only looking for responses from individuals who participated in round 1 of the questionnaire.

   ◯ Yes

   ◯ No (in this case you may stop here, and we thank you very much for your contribution at the workshop)

2. This consent form is a formal way of indicating that you agree to participate in this study. Please read the statements below and tick the boxes to show you agree:

   ☐ I understand that I am not obliged to give consent and I may withdraw my participation at any point of the process

   ☐ I understand that my contribution to the study will be recorded and used for research purposes

   ☐ I understand that the research from this study may be presented at research conferences or meetings

3. Please can you provide your email if you are happy for us to contact you about any feedback you give on the survey (e.g., for further clarification of points you have raised), and to invite you to participate in further rounds

   Enter your answer

What are the current priorities for UK equid welfare?

4. This question looks at <u>current welfare concerns</u> for UK equids.

   Participants in round 1 of the survey agreed that the following areas should be prioritised in order to improve UK equid welfare:

   - Obesity
   - High worm burdens
   - Basic needs (friends, forage, freedom to move) not being met
   - Weaning methods which cause stress/distress
   - Delayed euthanasia (defined as when an animal no longer has a good quality of life, and not euthanising sooner has resulted in prolonged and unnecessary suffering)
   - Insufficient veterinary care (e.g., veterinary care is delayed or not sought)
   - Confinement (e.g., equids in an enclosed space for a long time without areas to run freely)
   - Social isolation (e.g., equids not being able to see and/or touch other equids)
   - Riders being too heavy for their horses
   - Tack issues (e.g., poorly fitted tack causing pain or stress/distress, over-tacking)
   - Training approaches which stress/distress the equid or don't adequately prepare them for their career
   - Poor wellbeing of sport horses after their career (e.g., not rehomed appropriately, or kept in environments which don't meet their needs)
   - Equids being bred with poor conformation or inherited health issues

   They did not think that the following was important:

   - Inappropriate use of supplements (e.g., giving too many or not providing enough to meet the equid's needs)

   The welfare concerns on which they were undecided have been amended and are listed below, along with new concerns they identified. Please select how you think we should prioritise each of the suggested areas in order to improve UK equid welfare.

   Amendments and new statements are highlighted in **bold**.

   Guidance: In deciding the priority level, consider the total number of equids affected (i.e., the prevalence of the welfare concern), the impact it has on the welfare of individual affected equids (i.e., its severity), and how long that negative impact typically lasts (i.e., its duration).

	High priority	Medium priority	Low priority	Not a priority	Unsure/I don't know
Underfeeding **(e.g. not providing enough grazing, forage, supplements or calories for adequate nutrition)**	○	○	○	○	○
Poor welfare of **competition horses** during travel (e.g., long or frequent periods of travel, stress during travel)	○	○	○	○	○
Poor welfare **during transport to slaughter** (e.g., long or frequent periods of travel, stress during travel)	○	○	○	○	○
Physical injury (e.g. tendon issues) **or mental damage** sustained in training or competition	○	○	○	○	○
Incorrect matching of horses and **owners** (e.g., inexperienced **owners** with reactive horses)	○	○	○	○	○
**Early euthanasia (euthanasia of an animal with an acceptable quality of life, and which is not indicated for medical or behavioural reasons)**	○	○	○	○	○

5. Participants in round 1 of the survey were also undecided on whether the following welfare concerns should be prioritised:

   - Excessive veterinary intervention (e.g., invasive procedures that are not necessary and over-medication – including excessive use of joint medication)
   - Equids being bred with temperaments only suitable for limited purposes, which make them difficult to manage for the majority of equid owners

   In your opinion, is there anything we can change or add to these statements to make them clearer?

   Enter your answer

6. This question looks at factors contributing to current welfare concerns for UK equids.

   Participants in round 1 of the survey agreed that the following factors should be prioritised in order to improve UK equid welfare:

   - Owners/carers not understanding enough about health and disease management (e.g. body condition scoring, disease prevention and control)
   - Owners/carers not understanding enough about management (e.g. land management, appropriate housing of equids, use of appropriate tack)
   - Owners/carers not understanding enough about wellbeing (e.g. welfare and quality of life, building equine social groups)
   - Owners/carers not understanding equids' needs (e.g. the need for forage, friends, and freedom to move)
   - Owners/carers not able to recognise signs of stress/fear/pain
   - Lack of traceability of equids (e.g. inability to track a horse through its lifetime)
   - Lack of transparency around veterinary history (e.g. not sharing previous veterinary history)
   - Financial challenges for owners/carers (e.g. insufficient funds for full veterinary care, insufficient land for turnout)
   - Limited grazing availability in the UK (e.g. amount of grazing available across the UK or on some livery yards)
   - Poor biosecurity across the equine industry (e.g. insufficient strangles testing and alerting)

   The factors on which they were undecided have been amended and are listed below, along with new factors they identified. Please select how you think we should prioritise each of the suggested areas in order to improve UK equid welfare.

   Amendments and new statements are highlighted in **bold**.

   Guidance: In deciding the priority level, consider the total number of equids affected (i.e., the prevalence of the welfare concern), the impact it has on the welfare of individual affected equids (i.e., its severity), and how long that negative impact typically lasts (i.e., its duration).

	High priority	Medium priority	Low priority	Not a priority	Unsure/I don't know
Owners/carers being **unable to differentiate** between high-quality information (correct, evidence-based) and low-quality information	○	○	○	○	○
Owners/carers **not being willing to engage** with good-quality resources and education	○	○	○	○	○
Lack of a **single, comprehensive educational resource**	○	○	○	○	○
Lack of **freely available** high-quality educational resources	○	○	○	○	○
Lax rules on import of equids **which poses a risk for spread of disease and introducing new diseases into the UK**	○	○	○	○	○
Lax rules on export of equids **which leads to illegal export of horses from the UK to slaughter in Europe**	○	○	○	○	○
**Lack of a clearly defined and regulated breeding programme within the UK**	○	○	○	○	○

Humanising of equids by owners/carers (**attributing human emotions to them rather than keeping a horse-centric approach to their care, for example believing that horses are capable of respect**)	○	○	○	○	○
**Owners/carers having a poor ability to recognise and identify equid behaviours**	○	○	○	○	○
**Owners/carers having a poor understanding of how equids learn**	○	○	○	○	○
**Owners/carers lacking understanding or willingness to engage with evidence-based training methods**	○	○	○	○	○
**Owners/carers not acting on their knowledge of equine management (e.g. recognising fear/pain/stress behaviours but not acting on them – possibly due to cost, limited facilities, and/or unwillingness to change)**	○	○	○	○	○

**Owners/carers not being aware of their limitations and critical of their own knowledge and practices**	○	○	○	○	○
**Limited availability of non-grass turnout (e.g. dry lot or grass-free track system) for equids who cannot live out on grass**	○	○	○	○	○

How can we use artificial intelligence to solve the current welfare issues?

7. This question looks at areas where we could develop artificial intelligence (AI) to improve equid health and welfare.

Participants in round 1 of the survey agreed that the following areas should be prioritised for AI development:

- AI tools which monitor measures of normal behaviours and activity (e.g. temperature, eating, sleeping, and social interactions)
- AI tools which monitor indicators of positive or negative wellbeing
- AI tools which monitor signs of ill health/pain
- AI tools which monitor measures of wellbeing when ridden
- An AI tool which can combine different measures together to estimate quality of life for equids undergoing veterinary treatment
- AI tools which help equid carers (owners, trainers, staff, etc.) make decisions about when to call a veterinarian
- AI tools which give equid carers information about tack pressures and help make decisions about tack fit
- AI tools which help equid carers to determine body condition score
- AI tools which help equid carers to identify when an equid is lame
- AI tools which identify equids (for traceability)
- AI tools which identify significant health and welfare issues at slaughter
- AI tools which can monitor wellbeing, health, and behaviour for 24 hours/day
- AI tools which monitor equids 24 hours/day and send health alerts

The areas on which they were undecided have been amended and are listed below, along with new areas they identified. Please select how you think we should prioritise each of the suggested areas for AI development.

Amendments and new statements are highlighted in **bold**.

	High priority	Medium priority	Low priority	Not a priority	Unsure/I don't know
AI tools to help competition judges to rank entrants **(e.g. tools which make objective measurements about head position in dressage)**	○	○	○	○	○

AI tools which help equid carers (owners, trainers, staff, etc.) make decisions about wound care on the yard **(e.g. whether the wound is near a joint and veterinary attention should be sought)**	○	○	○	○	○
AI tools which help equid carers and health professionals make decisions about injury and rehabilitation plans **(e.g. by generating a recommended period of box rest and return to work plan post-injury)**	○	○	○	○	○
AI tools which digitise **diagnostic** procedures **which are time-consuming or laborious to perform manually** (e.g. faecal worm egg counting [FWEC], semen analysis)	○	○	○	○	○
AI tools which track and predict performance **(e.g. systems to automatically record and analyse competition data to develop predictions for likelihood of success for individual horses)**	○	○	○	○	○

AI tools which calculate risk profile for competitions **(e.g. systems which analyse weather and conditions data, compare to previous competitions and injury rates, and automatically generate predictions for risk of injury)**	◯	◯	◯	◯	◯
AI tools which filter and summarise current research **(e.g. systems which search databases for publications on specific topics, and generate AI summaries)**	◯	◯	◯	◯	◯
AI tools which monitor e quids 24 hours/day in veterinary hospi tals **(e.g. AI and camera systems which track and produce summaries on activities including colic signs, lameness, signs of foaling)**	◯	◯	◯	◯	◯

AI tools which monitor equids 24 hours/day to track normal behaviours **(e.g. camera systems which track and produce summaries on activities including sleeping, eating and passing droppings, or motion sensors which detect activity and distance covered at pasture)**	◯	◯	◯	◯	◯

8. Participants in round 1 of the survey were also undecided on whether the following area should be prioritised for development:

   - AI tools which help equid carers (owners, trainers, staff, etc.) make decisions about whether an equid needs a rug

   In your opinion, is there anything we can change or add to this statement to make it clearer?

   Enter your answer

9. This question looks at <u>how we can make AI tools accessible and useful.</u>

   Participants in round 1 of the survey agreed that the following would be effective ways to make AI tools accessible and useful:

   - Using celebrity endorsement
   - Writing guidelines in conversational language
   - Developing accessible websites
   - Putting it into an app
   - Making it simple to use
   - Making it possible to input data horse-side
   - Keeping the cost of equipment and use low
   - Having it integrated into competition rules by governing bodies (e.g. automating the checking of vaccination records)

The new ideas they identified are listed below. Please select how effective you think each suggestion would be for making a robust AI tool accessible and useful for horse owners/carers.

New statements are highlighted in **bold**.

	Very effective	Somewhat effective	Neither effective nor ineffective	Somewhat ineffective	Very ineffective	Unsure/I don't know
**Giving users of AI tools the choice between a more simple or more sophisticated user interface (to accommodate different levels of technological confidence, or to offer different functionality to owners and vets)**	○	○	○	○	○	○
**Making the AI simple to interpret**	○	○	○	○	○	○

10. Participants in round 1 of the survey were also undecided on whether the following ideas would be effective ways to make AI tools accessible and useful:

- Making it into a competition (i.e., users can see how their horse ranks against others)
- Making it into a game, offering people rewards

In your opinion, is there anything we can change or add to these statements to make them clearer?

Enter your answer

What are the potential problems and barriers to using AI to improve equid welfare?

11. This question looks at potential problems which could arise from using AI in equid welfare.

Participants in round 1 of the survey agreed that the following potential problem was a concern:

- Over-treatment (unnecessary or excessive medical interventions that do not benefit the equid or where the risks outweigh the potential benefits)

The potential problems on which they were undecided have been amended and are listed below, along with new problems they identified. Please select how concerned you are about each of these potential problems that were identified.

Amendments and new statements are highlighted in **bold.**

	Very concerned	Somewhat concerned	Neither concerned nor unconcerned	Somewhat unconcerned	Very unconcerned	Unsure/I don't know
Reduced human curiosity, **which reduces innovation and ideas**	◯	◯	◯	◯	◯	◯
Loss of **expert knowledge or knowledgeable individuals**	◯	◯	◯	◯	◯	◯
Loss of **expert evaluation or the ability to evaluate**	◯	◯	◯	◯	◯	◯
**Owners/carers becoming complacent**	◯	◯	◯	◯	◯	◯

**Fewer interactions between equids and humans**	○	○	○	○	○	○
**Over-reliance on AI tools for equid management (to the extent that if the AI failed, it would leave equids without adequate care)**	○	○	○	○	○	○
**Automation leading to loss of respect and job value for skilled grooms and yard managers**	○	○	○	○	○	○
**Loss of implicit knowledge from the industry**	○	○	○	○	○	○

12. Participants in round 1 of the survey were also undecided on whether the following potential problem was a concern:

    - Under-treatment (insufficient or inadequate treatment, which fails to provide the necessary care to the equid)

    In your opinion, is there anything we can change or add to this statement to make it clearer?

    Enter your answer

13. This question looks at potential barriers to the **development** of AI tools for equid welfare.

    Participants in round 1 of the survey agreed that solving the following barriers should be a priority in order to improve equid welfare:

    - Poor data quality to train models

- Poor model accuracy
- Bias in how data are collected
- Difficulties validating the AI
- Lack of transparency

The potential barriers on which they were undecided have been amended and are listed below, along with new barriers they identified. Please select how you think we should prioritise solving each of these barriers in order to improve equid welfare.

Amendments and new statements are highlighted in **bold**.

	High priority	Medium priority	Low priority	Not a priority	Unsure/I don't know
Bias towards commercially viable interests **affecting how algorithms are developed**	○	○	○	○	○
Bias in how the algorithm is trained and interpreted **due to low quality data/poor algorithm development**	○	○	○	○	○
Concerns regarding ownership of the information generated **and who your personal data is shared with**	○	○	○	○	○
The general public finding it difficult to understand and critically assess AI systems (e.g. how is AI using the data, **how accurate or reliable the data are**)	○	○	○	○	○
Lack of anonymity **(e.g. sharing your personal data with the company)**	○	○	○	○	○

**Limited current understanding of certain equid behaviours (e.g. facial expressions)**	○	○	○	○	○
**Lack of agreement as to which behaviours indicate optimal welfare in an individual equid**	○	○	○	○	○
**Difficulty developing more complex AI tools (e.g. a tool to identify equid facial expressions of pain)**	○	○	○	○	○
**Unethical collection of data to train AI models**	○	○	○	○	○

14. This question looks at potential barriers to the **use** of AI tools for equid welfare.

Participants in round 1 of the survey agreed that solving the following barriers should be a priority in order to improve equid welfare:

- The need for careful interpretation
- The need to cross-check AI's output with other sources
- Cost
- Issues regarding responsibility of decisions guided by AI
- Client dissatisfaction/lack of trust in AI

The potential barriers on which they were undecided have been amended and are listed below, along with new barriers they identified. Please select how you think we should prioritise solving each of these barriers in order to improve equid welfare.

Amendments and new statements are highlighted in **bold**.

	High priority	Medium priority	Low priority	Not a priority	Unsure/I don't know
Environmental impact of the AI **(e.g. energy consumption required to run computer systems with large data sets, environmental impacts of generating and disposing of hardware systems)**	○	○	○	○	○
**Practical challenges of using AI to monitor behaviour (e.g. the challenge of using static cameras to monitor horses living out in the field)**	○	○	○	○	○

How can we solve these problems?

15. This question looks at proposed solutions to how we can help AI to effectively improve equid welfare.

Participants in round 1 of the survey agreed that the following solutions would help AI to improve equid welfare:

- Have an approval system for equid welfare AI systems
- Share data between groups that can work together (e.g. AI companies)
- Anonymise data for equid welfare AI systems
- Make sure that the data used in equid welfare AI systems are not biased
- Make models for equid welfare AI systems using high quality data
- Treat equid welfare AI systems as supporting evidence and monitoring tools, rather than tools which offer a definitive answer
- Provide education on how to interpret the output of equid welfare AI systems
- Ensure human interaction is maintained in diagnoses (e.g. A human is always involved in the diagnosis provided)
- Have a regulatory group certifying equid welfare AI tools
- Collaborate across industries

The proposed solutions on which they were undecided have been amended and are listed below, along with new solutions they identified. Please select whether you agree, disagree or are neutral for each of the statements below relating to suggested solutions.

Amendments and new statements are highlighted in **bold**.

	Strongly agree	Agree	Neutral	Disagree	Strongly disagree	Unsure/I don't know
Have legislation to regulate equid welfare AI systems **to protect personal data and reduce bias**	○	○	○	○	○	○

**Prioritise AI tools which involve long-term monitoring (to assess welfare and/or guide management decisions) rather than tools which use snapshots of information (to guide diagnosis)**	○	○	○	○	○	○
**Prioritise areas where change is more likely/achievable (e.g. where change does not require legislation)**	○	○	○	○	○	○
**Ensure that AI tools tell users when they are making inferences based on a limited knowledge base (e.g. when interpreting equid facial expressions)**	○	○	○	○	○	○
**Prioritise areas which are better understood, or which have a more objective outcome (e.g. prioritising tools which monitor sleep rather than tools which estimate equid welfare)**	○	○	○	○	○	○
**Make sure AI models are evidence-based**	○	○	○	○	○	○

**Have codes of best practice to guide the use of equid welfare AI systems**	◯	◯	◯	◯	◯	◯

Comments

16. Are there any comments you would like to make about the survey, or any of the ideas within it?

Enter your answer

Questionnaire round 3: Use of artificial intelligence to improve equid health and welfare

Thank you for taking the time to complete round 2 of this questionnaire. We really appreciate the time you have given throughout the project and it is exciting to see such clear priorities for the use of AI for equid welfare taking shape.

We have now reviewed the results, identified the few remaining statements about which participants had mixed views (i.e. 25-75% of people thought that a certain welfare concern/concern about AI was important), and taken on board all of the participants' feedback.

In this final round of the survey, **we would really appreciate your input to help us identify which of these updated/new statements are important priorities going forward**. As previously, this questionnaire asks you to prioritise or indicate your agreement on a variety of statements.

When you submit this form, it will not automatically collect your details like name and email address unless you provide it yourself.

Definition of terms, consent form, and further contact

Key terms – Please note that throughout this survey we use the term "equid" to include horses, ponies, donkeys, and their hybrids.

Consent form – You must complete this form in order to complete this survey.

Participation in this research is entirely voluntary and there is no obligation to take part. Please note you must be over 18 years of age to participate. This study has been approved by the University of Nottingham, School of Veterinary Medicine and Science Ethics Committee. Further information can be obtained by contacting the research team at sarah.freeman@nottingham.ac.uk.

As a participant in this study you can:

- Request to see a copy/summary of the completed study
- Request to see any information written down/kept during the process of data collection

1. Did you complete round 2 of this questionnaire (titled "Use of artificial intelligence to improve equid health and welfare", available between June 16th and July 3rd)?

   Due to our methodology we are only looking for responses from individuals who participated in round 2 of the questionnaire.

- ○ Yes
- ○ No (in this case you may stop here, and we thank you very much for your contributions)

2. This consent form is a formal way of indicating that you agree to participate in this study. Please read the statements below and tick the boxes to show you agree:

- ☐ I understand that I am not obliged to give consent and I may withdraw my participation at any point of the process
- ☐ I understand that my contribution to the study will be recorded and used for research purposes
- ☐ I understand that the research from this study may be presented at research conferences or meetings

3. Please can you provide your email if you are happy for us to contact you about any feedback you give on the survey (e.g., for further clarification of points you have raised)

Enter your answer

What are the current priorities for UK equid welfare?

4. This question looks at current welfare concerns for UK equids.

   In round 2 of the survey, participants were undecided on the importance of certain welfare concerns. These have been amended and are listed below, along with a new concern that they identified.

   Please select how you think we should prioritise each of the suggested areas in order to improve UK equid welfare.

   Amendments and new statements are highlighted in **bold**.

   Guidance: In deciding the priority level, consider the total number of equids affected (i.e., the prevalence of the welfare concern), the impact it has on the welfare of individual affected equids (i.e., its severity), and how long that negative impact typically lasts (i.e., its duration).

	High priority	Medium priority	Low priority	Not a priority	Unsure/I don't know
**Welfare issues associated with artificial insemination (e.g. inappropriate selection of mares for breeding, the invasive procedures involved, and the stress of veterinary hospital stays)**	○	○	○	○	○

Excessive veterinary intervention **to keep horses competing and training at high levels, or to maintain horses with poor health or conformation in work (e.g. use of joint medication when retirement or reducing workload or level of competition may be more beneficial for the horse's long-term health)**	○	○	○	○	○
Vets undertaking invasive procedures **without clear justification (e.g. additional diagnostic procedures when they will not change the treatment or outcome, or surgical intervention when an evidence-based decision indicated a medical option was preferable)**	○	○	○	○	○
Equids being bred with **physical characteristics (e.g. big movement)** which make it difficult for the majority of owners **to effectively support their physical and mental development**	○	○	○	○	○

Equids being bred **for their physical characteristics and abilities without sufficient consideration of temperament and handleability for them to be suitable for majority of equid owners to manage**	○	○	○	○	○

How can we use artificial intelligence to solve the current welfare issues?

5. This question looks at how we can make AI tools accessible and useful.

   In round 2 of the survey, participants were undecided on how effective certain suggestions would be at making AI tools accessible and useful. These have been amended and are listed below.

   Please select how effective you think each suggestion would be for making a robust AI tool accessible and useful for horse owners/carers.

   Amendments are highlighted in **bold**.

	Very effective	Somewhat effective	Neither effective nor ineffective	Somewhat ineffective	Very ineffective	Unsure/I don't know
**Creating targets or challenges for owners to consider and aim for (e.g. a monthly challenge for your horse to have a good night's sleep)**	○	○	○	○	○	○
**Linking to commercial incentives (e.g. insurance company discounts if specific monitoring or care requirements are met, or discounted entry fees if AI registration systems are used)**	○	○	○	○	○	○

What are the potential problems and barriers to using AI to improve equid welfare?

6. This question looks at potential problems which could arise from using AI in equid welfare.

   In round 2 of the survey, participants were undecided on whether certain potential problems associated with use of AI in equid welfare were of concern. One of these has been amended and is listed below.

   Please select how concerned you are about the following potential problem which was identified.

   Amendments are highlighted in **bold.**

	Very concerned	Somewhat concerned	Neither concerned nor unconcerned	Somewhat unconcerned	Very unconcerned	Unsure/I don't know
Under-treatment (insufficient or inadequate treatment **from healthcare professionals**, which fails to provide the necessary care to the equid)	○	○	○	○	○	○

Comments

7. Are there any comments you would like to make about the survey, or any of the ideas within it?

Enter your answer
